# Seasonal variation of femoral fractures in the state of São Paulo, Southeast Brazil

**DOI:** 10.11606/s1518-8787.2019053001051

**Published:** 2019-08-12

**Authors:** Mônica Marin de Souza, Eniuce Menezes de Souza, Altacílio Aparecido Nunes, Edson Zangiacomi Martinez

**Affiliations:** I Universidade Estadual de Maringá. Maringá, PR, Brasil; II Universidade de São Paulo. Faculdade de Medicina de Ribeirão Preto. Ribeirão Preto, SP, Brasil

**Keywords:** Femoral Fractures, epidemiology, Risk Factors, Seasons, Fourier Analysis, Ecological Studies

## Abstract

**OBJECTIVE:**

To examine the effect of seasonality on femoral fracture incidence among people residing in the state of São Paulo, Brazil.

**METHODS:**

Ecological study based on a consecutive series of 216,348 reports of hospital admissions caused by femoral fractures. A Bayesian statistical model was used for time series analysis, considering the monthly average number of events of femoral fractures per day as a dependent variable.

**RESULTS:**

Among the female population, significant seasonal effects were observed only for older women, aged 60 years or more. Among younger men (aged less than 20 years) there is not a clear seasonal effect, but among the other age groups there seems to exist a higher number of cases of femoral fractures during the coldest months of the year.

**CONCLUSIONS:**

In general, more cases of fractures occur during the coldest months of the year; however, men and women have different patterns of incidence according to each age group.

## INTRODUCTION

Femoral fractures are a common cause of mortality and functional loss, mainly among older people^1^^,^^2^. Osteoporosis is one of the main risk factors for these fractures^3^, and other known risk factors are falls, early menopause, and sedentary lifestyle^1–4^. It is estimated that the life expectancy of patients suffering from femoral fracture is reduced from 15% to 20%^4^. A review study showed that, in Brazil, 5.5% of patients with fracture in the proximal third of the femur die during hospitalization and 4.7% die at the end of one month of follow-up^5^. The cost per femoral fracture surgery is significant to the Brazilian health system, as the direct cost of hospitalization is estimated between 4,100 and 70,000 dollars. Furthermore, the hospital medical resources used in the postoperative period accounted for 5.7% of the direct cost of hospitalization^6^.

A number of authors has studied the effects of seasonality on femoral fracture incidence^7–16^. According to a prospective study including 3,034 consecutive hip fracture patients in the United Kingdom, more fractures occurred during the winter compared to summer, and a tendency towards higher mortality for those patients admitted in the winter months^7^. However, this study did not show significant difference in patient characteristics between the winter and summer seasons^7^. Another study^8^ that considered the monthly number of operated proximal femoral fractures across 31 hospitals of North-West England and Scotland described an increase in the incidence of hip fractures during the months of December and January (winter months in the northern hemisphere). Douglas et al.^9^ studied the seasonal variation of hip fracture admissions at three different latitudes: Scotland, Shatin (Hong Kong), and Auckland (New Zealand). These authors also concluded that there is a higher incidence of fractures during the coldest months.

Considering the fact that most of the articles on the effects of seasonality on fracture incidence includes populations from countries of the Northern Hemisphere, the objective of this article is to describe the seasonal variation of femoral fractures in the state of São Paulo, Brazil.

## METHODS

This is an ecological study, conducted over a period from January 2008 to December 2017. A total of 216,348 reports of hospital admissions caused by femoral fractures was considered. This number corresponds to approximately one quarter of the admissions reported throughout the country in this period. São Paulo is the most industrialized state in Brazil, with a population of approximately 45 million inhabitants in an area of 248,209 square kilometers (according to official statistics of the Brazilian Institute of Geography and Statistics, available in https://cidades.ibge.gov.br/brasil/sp/panorama). The territory is situated between the latitudes 19°46S and 25°18S. Data on the fracture incidence were obtained from the Hospital Information System (SIH), available from the DATASUS (Database of the Brazilian Unified Health System). The SIH includes all hospitalizations within the Unified Health System (SUS). Yearly population projections based on census data were obtained with use of the TABNET system, also available from DATASUS (http://www2.datasus.gov.br/DATASUS/index.php).

This study was conducted exclusively with secondary data of public access, without identification of subjects, and its procedures are in accordance with the principles of ethics in research involving human beings. Thus, the study was waived from formal review and informed consent by the institutional Research Ethics Committee. However, the study was conducted according to the Helsinki declaration and good clinical practice.

Let *y*_*t*_ be the number of cases of femoral fractures recorded at the month *t* in the state of São Paulo, divided by the number of days of the corresponding month. Thus, *y*_*t*_ refers to the monthly average number of events of femoral fractures per day. Considering the period from January 2008 to December 2017, we have *t* = 1, 2,…,120. The statistical data analysis was based on the time series model given by the expression

$$y_{t}=\beta_{0}+\beta_{1}t+S(t)+e_{t},$$

where β_0_ is an intercept, β_1_ describes the trend component of the time series data, *S*(*t*) is a function related to the seasonality of the time series, and the terms *e*_*t*_ follow an autoregressive structure of order 1, given by *e*_*t*_ = *ρε*_*t*–1_ + *ε*_*t*_. We assumed that the term *ε*_*t*_ follows a normal distribution with mean zero and variance *σ*^2^_*ε*_. The seasonality of the time series is given by

$$S(t)=\eta_{1}\sin\left(\frac{2\pi t}{12}\right)+\eta_{2}\cos\left(\frac{2\pi t}{12}\right).$$

Thus, the significance of the terms *η*_1_ and *η*_2_ determine the significance of the seasonal pattern of the time series and its amplitude can be obtained from √*η*_1_^2^ + *η*_2_^2^. This model formulation is based on the well-known Prais-Winsten method, commonly used in epidemiological studies^17^^,^^18^. Estimates of the parameters of interest were obtained by Bayesian methods using the Gibbs sampler. Non-informative prior distributions were used, and posterior distributions of the parameters were constructed from 500,000 simulations after a burn-in period of 10,000 simulations (initial iterations to allow simulations to stabilize).

The adequacy of the model to describe the data includes a residual analysis, where residuals (differences between the observed values for *y*_*t*_ and the correspondent values predicted by the model) should be a stationary time series, with non-significant serial correlation between their successive values. These assumptions were verified by graphs of the partial autocorrelation function (not showen in this article) and using the augmented Dickey-Fuller test. We used OpenBUGS software (freely available at http://openbugs.net) for all statistical analyses and the R language version 3.4.3 (freely available at https://www.r-project.org/) to generate all graphs and tables^19^.

## RESULTS

The incidence of femoral fractures according to age groups and sex are shown in Table 1, considering the years of 2008, 2011, 2014, and 2017, with the rates per 100,000 population. These rates were obtained directly from the data and without the use of the proposed regression model. We can observe that the fracture incidence is higher among men than women, considering the age groups up to 60 years. However, after the age of 70 years, the incidence is much higher among women than among men. For each age group, there are no significant differences between the incidences when compared between all four years described in Table 1.

**Table 1:** Incidence of femoral fractures in man and women per 100,000 population per year, state of São Paulo, Brazil, years of 2008, 2011, 2014 and 2017.

Age groups (years)	Men				Women			
	
	2008	2011	2014	2017	2008	2011	2014	2017
0–4	19.5	19.1	21.8	19.8	10.9	9.2	10.3	9.4
5–9	14.8	12.4	13.9	11.6	7.7	8.0	6.9	6.9
10–14	22.0	22.0	18.9	17.5	8.9	9.2	6.3	7.2
15–19	45.7	54.2	53.5	46.8	12.9	12.0	12.1	12.1
20–29	56.9	59.7	57.4	54.4	9.2	11.9	10.3	11.0
30–39	32.5	39.0	37.6	34.0	6.3	6.5	6.2	8.1
40–49	36.8	36.9	36.0	37.5	8.9	9.5	7.5	8.7
50–59	48.0	49.8	50.8	53.6	23.2	25.9	21.0	23.3
60–69	90.0	86.2	74.9	86.8	83.7	81.3	72.1	82.1
70–79	200.0	202.8	179.9	188.3	306.0	306.5	286.8	300.8
80 or more	594.4	550.7	496.2	553.1	1,007.5	981.5	901.0	941.7
***								***
Total	50.0	53.2	52.0	54.3	43.0	46.8	45.9	53.4

**Figure 1:**
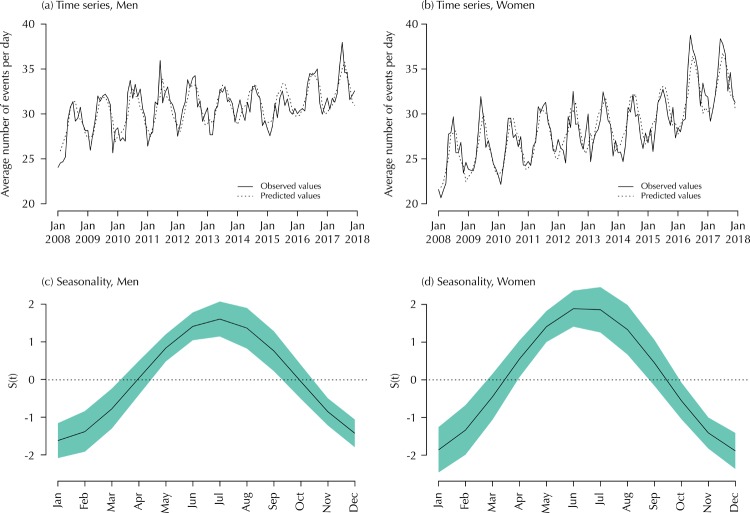
Panels (a) and (b): observed and predicted time series describing the monthly average number of events of femoral fractures per day, from January 2008 to December 2017, in the state of São Paulo, Brazil. Panels (c) and (d): graphs for the seasonality function *S*(*t*) of the time series, according to the months of femoral fractural occurrence. The green areas correspond to Bayesian credible regions.

Panels (a) and (b) in Figure 1 compare the observed time series for the monthly average number of events of femoral fractures per day and the values estimated from the proposed Bayesian model. The model was separately fitted to data for both sexes. It is possible to observe that the predicted values are close to the observed values, suggesting a reasonable fit of the model to the data in both cases. We also fitted alternative models for the data while considering that the *e*_*t*_ terms follow an autoregressive structure of order 2, but we did not observe an expressive improvement in the fit compared to the previous models (comparisons between models were based on the deviance information criterion).

Bayesian estimates for the model parameters are shown in Table 2, with their correspondent 95% credible intervals. The 95% credible intervals are the Bayesian equivalent of standard confidence intervals, representing the central 95% of the range of values that are credible for the parameter estimated. While the values for the *β*_0_ parameter are interpreted as hypothetical means when *t* = 0 (a “starting point” for the series), the values for *β*_1_ are positive for both sexes, indicating an increasing trend over time. This increase is probably a result of the process of population aging ocurring in the state of São Paulo during the studied period. As intuitive visual inclinations in Figure 1, the estimated increasing trend for women is a bit stronger than for men. The 95% credible intervals for *η*_1_ and *η*_2_ do not include zero, indicating a significant seasonal effect for both time series. Credible intervals for the autoregressive coefficient *ρ* also do not include zero, indicating a significant and positive correlation between successive values of *e*_*t*_ in the time series.

**Table 2:** Bayesian estimates for the model parameters, including the monthly average number of events of femoral fractures per day, period from January 2008 to December 2017, state of São Paulo, Brazil.

Parameter	Men		Women	
	
	Estimate	95%CI	Estimate	95%CI
β_0_	28.98	(28.16–29.80)	24.07	(22.96–25.16)
β_1_	0.029	(0.017–0.041)	0.073	(0.057–0.088)
η_1_	-1.540	(-2.026– -1.036)	-1.502	(-2.127– -0.881)
η_2_	-1.655	(-2.145– -1.164)	-2.463	(-3.084– -1.853)
ρ	0.356	(0.175–0.536)	0.415	(0.241–0.593)

Panels (c) and (d) in Figure 1 show graphs for the seasonality function *S*(*t*) of the time series, according to the months during which femoral fractures occurred. The graphs evidence that a higher number of fracture cases were reported between June and August, the coldest months in the Southern Hemisphere. The green areas in the graphs correspond to Bayesian credible regions, which is the interval in which we are 95% sure the true curves lie.

**Figure 2:**
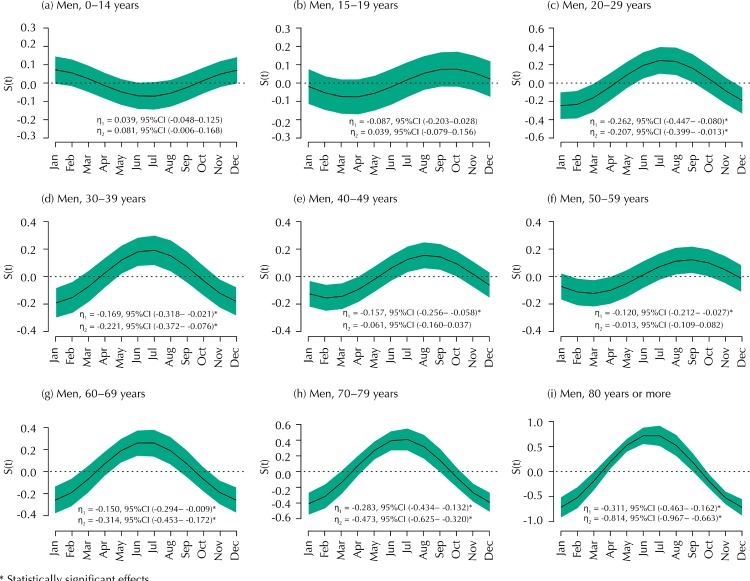
Graphs for the seasonality function *S*(*t*) of the time series, according to the months of femoral fracture occurrence, stratified by age groups and considering the male population. The green areas correspond to Bayesian credible regions.

Figure 2 shows plots of the seasonal effects given by the function *S*(*t*) stratified by age groups and considering the male population. In each graph, when the horizontal line crossing the zero is entirely within the Bayesian credible region, we also have an indicative that the seasonal effect is not significant. Note that it was not possible to standardize the y-axis scales of these plots, which would impair the visualization of curve behavior. Therefore, we have not found significant seasonal effects for the age groups 0–14 and 15–19 years. Unlike other age groups, where a higher number of fracture cases were reported between June and August, it was observed a higher incidence between August and September among men aged 40–49 and 50–59 years.

* Statistically significant effects.

**Figure 3:**
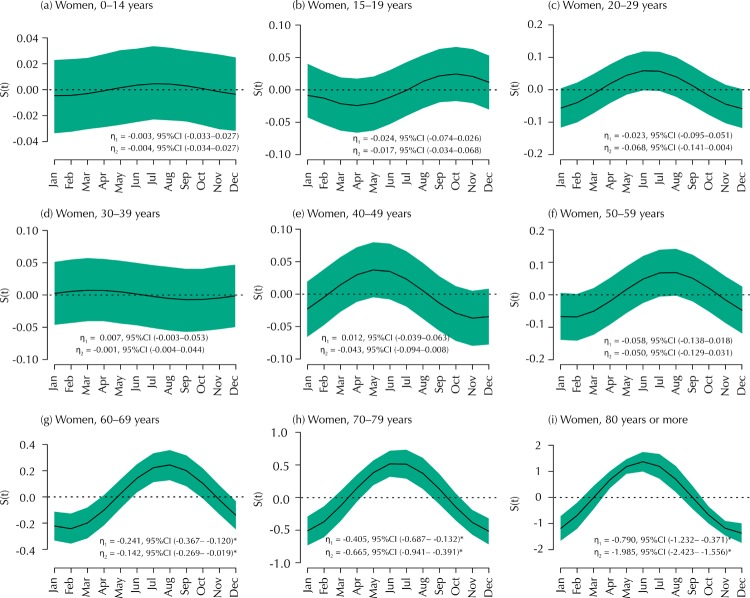
Graphs for the seasonality function *S*(*t*) of the time series, according to the months of femoral fracture occurrence, stratified by age groups and considering the female population. The green areas correspond to Bayesian credible regions.

Figure 3 shows plots of the seasonal effects given by the *S*(*t*) function stratified by age groups and considering the female population. We observe significant seasonal effects only for older women aged 60 years or more. Among the age group 60–69, more cases of fractures were reported between July and September, while a higher incidence was observed between May and August among women aged 70–79 and 80 years or more.

* Statistically significant effects.

## DISCUSSION

The results shown in Table 1 show that the incidence of femoral fractures among men increased steadily with age, up to the age group of 20–29 years (54.4 cases per 100,000 in 2017). It is possible that men of this age group are more subject to risks due to activities related to work and sports. The incidence among men decreased in the age group of 30–39 years and again increased steadily with age, up to the age group of 80 years or more. Incidence of femoral fractures was lower in women than in men before the age group of 60–69 years. In the age group of 60–69 years the incidence is similar between men and women, and after the age of 70 years, the incidence is much higher among women than among men. The number of fractures that are due to osteoporosis can explain these differences^3^.

In the time series analysis using the Bayesian model, results in Table 2 and Figure 1 (Panels (c) and (d)) shows an important seasonal variation in the incidence of femoral fractures with a higher incidence during the coldest months compared to the summer period. This supports findings from several other studies^7–16^ although reasons for this variation remain uncertain. Among possible hypotheses, Douglas et al.^9^ state that winters are darker as well as colder, and this may add to the number of falls in this season. While Ralis^15^ attributed the fractures to slipping on ice and snow, other authors describe that many cases in the winter time occur in indoor environments. Bastow et al.^20^ proposed that undernutrition of older people is a risk factor for hypothermia in cold weather, and hypothermia could lead to impairment of judgement and co-ordination, and consequently, to injury. Chiu et al.^21^ postulated that the hindrance of free movements and increased clumsiness of patients with many layers of clothes put on during the cold weather might be associated to an increase of the incidence of fractures. The study from Mazzucchelli et al.^22^ showed a short-term association between several indicators of air pollution and daily hospital admissions due to osteoporotic fracture, which can also in part explain the seasonal variations in the numbers of femur fractures. Other articles support the role of the low exposure to sunshine in the vitamin D deficiency^23^. According to this hypothesis, the low serum concentration of 25-hydroxyvitamin D leads to a low 1,25-dihydroxyvitamin D concentration and then to a higher serum parathyroid hormone concentration, especially during the winter season^23^. On the other hand, a low level of parathyroid activity can be associated with high bone turnover, leading to cortical bone loss and low density bone. Lastly, this effect may lead to fracture^23^.

Results in Figures 2 and Figure 3 demonstrate the strong effect of sex and age groups on the seasonal patterns of the time series. Figure 2 shows that among younger men (aged less than 20 years) there is not a clear seasonal effect, but among the other age groups there seems to exist a higher number of cases of femoral fractures in the coldest months of the year. On the other hand, results in Figure 3 show that women sustaining femoral fractures during the coldest months were older (aged more than 60 years) and probably less active. Again, it is unclear what mechanism may exist to elicit this phenomenon with precision, as many of the probable reasons for femoral fracture, mentioned in the previous paragraph, may also act in this scenario. So, future research conducted in specific patient populations may include more variables that best describe these cases, such as the environment in which the fracture occurred (outdoor or indoor), the form of the accident (e.g. falls, traffic accident, sports accidents, stumbling on an object), the severity of the trauma, among others.

If we observe the results in Table 1, Table 2, and Figure 1 (Panels (a) and (b)) for men and women, the estimated average of fractures per day at the beginning of the analysis (January 2008) was almost 29 (95% credible interval from 28.16 to 29.80) and about 24 (95% credible interval from 22.96 to 25.16). After that, a stronger trend for woman (0.073 which means an increase of about one case each 14 days) than for man (0.029 meaning an increase of about one case each 35 days) has conducted this group to reach high rates of fractures per day in the last two years. Furthermore, the contribution of the seasonal term, expressed by its amplitude, is slightly stronger for women (2.88) than for men (2.26), although, on average, that is less evident, as shown in Figure 3.

A potential limitation of this study is the underreporting of the events and the possibility of femoral fracture miscoding. The Hospital Information System (SIH) has incomplete coverage and there are uncertainties about the reliability of its data^24^. It covers only records obtained from the hospitals affiliated to the Brazilian Unified Health System (SUS), excluding the admissions from the Private Health System (PHS) and the Supplementary Health System (SHS), which are administrated by private healthcare insurance companies or health cooperatives. Moreover, due to its ecological design, we cannot draw causal inferences about the effects of the different seasons of the year on fracture incidence. However, despite its descriptive nature, findings of our study are useful for better understand how fracture occurrences are distributed over time. This information is useful for the future planning of health care.

In conclusion, more cases of fractures occur in the coldest months of the year; however, men and women have different patterns of incidence according to each age group.
